# Investigation of intensive care nurses’ moral intelligence and their attitudes toward medical errors: a cross-sectional study

**DOI:** 10.1186/s12912-026-05039-3

**Published:** 2026-07-16

**Authors:** Burcu Kübra Süha

**Affiliations:** Faculty of Health Science, Division of Nursing, Sivas Cumhuriyet University, Sivas, 58140 Turkey

**Keywords:** Moral intelligence, Medical error, Nursing, Patient safety, Intensive care

## Abstract

This study aims to determine the relationship between the moral intelligence of intensive care nurses and their attitudes towards medical errors. The study was designed as a descriptive, cross-sectional study. The study sample consisted of 158 nurses working in intensive care units at a university hospital. Data were collected using a personal information form and two scales: the Yakut Moral Intelligence Scale and the Attitude towards Medical Errors Scale. Descriptive statistics, an independent samples t-test, a one-way analysis of variance (ANOVA) and a Pearson correlation analysis were used to analyse the data. The results showed that nurses’ attitudes towards medical errors were moderately high (mean score: 3.72 ± 0.70), and their moral intelligence scores were moderate (mean score: 79.53 ± 11.47). No significant differences were found according to gender or educational level (*p* > .05). A strong positive significant correlation was found between moral intelligence and attitudes towards medical errors (*r* = .850, *p* < .01). The findings suggest that higher moral intelligence is associated with more positive attitudes toward medical errors and may contribute to strengthening patient safety culture.

## Introduction

Moral intelligence is the awareness and utilisation of moral capacities during the cognitive process that precedes behaviour, whereby actions are filtered through mental and ethical evaluations. This concept provides a guiding framework, particularly for individuals in managerial or leadership positions, for addressing workplace challenges and decision-making processes [1, 2]. The scientific literature widely acknowledges that, before engaging in any action, the mind operates a decision-making mechanism involving multiple regions of the brain [1–3].

Moral intelligence is a skill encompassing the ability to distinguish right from wrong, make ethical decisions and act on them. Those with higher levels of moral intelligence are believed to evaluate their choices cognitively before acting, determining whether a behaviour is right or wrong. This evaluative process guides immediate actions and informs future decisions based on the outcomes of those actions [3–5].

Furthermore, moral intelligence is closely associated with ethical sensitivity, professional responsibility and integrity, particularly in healthcare settings where decisions directly affect patient outcomes [5–7]. In this context, developing moral intelligence is crucial for fostering ethical awareness and improving the quality and safety of healthcare services.

Recent literature further conceptualises moral intelligence as a multidimensional construct that encompasses integrity, responsibility, compassion and forgiveness. These qualities are all essential for ethical decision-making and professional conduct [8, 9]. In healthcare settings, moral intelligence is gaining increasing attention due to its critical role in guiding clinical judgements, supporting ethical sensitivity and promoting patient-centred care [6, 10]. Furthermore, research suggests that higher levels of moral intelligence are associated with greater professional accountability, more effective management of ethical dilemmas, and improved quality of care [11, 12].

Therefore, moral intelligence is a vital skill for healthcare professionals, enabling them to integrate ethical principles into their practice and ensuring that their actions align with professional standards and universal moral values. Previous studies have shown that individuals who possess and effectively apply moral intelligence competencies tend to establish more transparent, trust-based and constructive relationships in the workplace [13, 14].

Nurses are the main professional group responsible for providing continuous patient care within healthcare systems. Nursing care encompasses fundamental practices aimed at maintaining, improving and restoring patients’ health and well-being. People may need professional care and support at different times in their lives, which is why care is such a key concept in nursing education, healthcare systems and the legal and ethical frameworks that govern the profession [14, 15]. Respecting oneself and others as individuals with intrinsic value is a core requirement of moral intelligence. Consequently, nursing’s primary objective is to provide care that upholds human dignity and rights [16, 17].

One of the major challenges faced by healthcare systems is the presence of ethical dilemmas [18]. In clinical practice, nurses often encounter ethical issues that require careful judgement and decision-making. Ethical practice in professional nursing requires the recognition and application of ethical principles in all nurse–patient interactions and interventions [19]. However, the ability to understand and implement these principles is closely related to human intelligence. Intelligence is broadly defined as the capacity for thinking, learning and adapting to new situations. It encompasses multiple dimensions, including cognitive, emotional, spiritual and moral intelligence [12, 20].Moral intelligence, in particular, involves the acquisition and application of ethical rules and principles, reflecting an individual’s ability to process and manage ethical issues effectively [21].Therefore, nurses demonstrate their moral intelligence competencies when addressing ethical challenges encountered during patient care. Integrating moral intelligence into nursing practice may support ethical decision-making and professional conduct and may contribute to improved patient experiences and care delivery efficiency.

Despite the aim to eliminate errors in healthcare delivery, the complexity of healthcare systems and the multidisciplinary nature of care provision mean that medical errors are inevitable. These errors are considered a key indicator of patient safety [22, 23]. The Joint Commission defines medical errors as the result of inappropriate or unethical actions by healthcare professionals, or negligence and inadequacy in professional practice, which result in harm to the patient. Even when no harm occurs, deviations from standard care processes are also classified as medical errors [24].

Studies indicate that physicians are responsible for around 62.1% of medical errors, whereas nurses account for approximately 10% [25]. Among patients affected by medical errors, 45.6–49.4% die, while 31.4% become permanently disabled [26]. Recent studies indicate that medical errors remain a common challenge in nursing practice, particularly in high-risk clinical settings. Communication breakdowns, excessive workload, staff fatigue, burnout, inadequate handovers, and system-related factors have been identified as major contributors to medical errors and patient safety incidents among nurses [27]. Even when patient harm does not occur, such errors can significantly impact patient trust and satisfaction [28, 29]. Furthermore, complaints against nurses are often associated with inadequate communication, incomplete documentation, failure to advocate for patients, substandard care and insufficient monitoring, with negligence being the most common legal issue [24, 28]. To improve patient safety and reduce healthcare-related harm, it is essential to identify the root causes of medical errors, determine the factors affecting error reporting, and develop institutional policies and preventive strategies [23, 30]. Failure to recognise and report medical errors hinders the development of effective prevention strategies. Attitudes, formed through the integration of cognition, emotion and behavioural tendencies, play a critical role in shaping perceptions of medical errors [31]. Therefore, it is crucial to determine the attitudes of healthcare professionals towards medical errors, particularly those of nurses who provide continuous 24-hour care, in order to increase awareness, improve reporting rates, and ultimately contribute to reducing medical errors [22, 32].

Although previous studies have examined nurses’ moral intelligence and attitudes toward medical errors separately, the potential relationship between these variables remains largely unexplored. Moral intelligence may influence nurses’ attitudes toward recognising, reporting, evaluating and preventing medical errors because ethical awareness, responsibility, empathy and self-regulation are essential components of safe clinical practice.

Nurses with higher moral intelligence may be more sensitive to patient safety issues and may demonstrate more positive attitudes toward recognising, reporting, and preventing medical errors. However, evidence examining the relationship between moral intelligence and attitudes toward medical errors is limited. Therefore, this study aimed to investigate the relationship between moral intelligence and attitudes toward medical errors among intensive care nurses.

## Materials and methods

### Study design

This study was designed as a descriptive, cross-sectional study.

### Setting and characteristics of the study

The study was conducted in the intensive care units at Sivas Cumhuriyet University Health Services Research and Application Hospital. The hospital has a total of ten such units, including an ICU for internal medicine, a chest diseases ICU, a neurology ICU, a neurosurgery ICU, a general surgery ICU, a neonatal ICU, an obstetrics and paediatrics ICU, a coronary ICU, a cardiovascular surgery ICU and an anaesthesia ICU. These units are specialised clinical settings where specific treatment and care practices are implemented.

Nurses working in intensive care units are exposed to fast-paced and demanding working conditions. The number of nurses in each unit varies depending on patient numbers and bed capacity.

### Study population and sample

The study population consisted of 250 nurses working in the intensive care units of Sivas Cumhuriyet University Health Services Research and Application Hospital. The minimum sample size was calculated using the formula for a known population with a 95% confidence level, 5% margin of error, and an assumed prevalence of 50% (*p* = .50, q = 0.50). The required sample size was determined to be 152 nurses. A convenience sampling method was used, and all eligible nurses were invited to participate in the study. Of the 250 nurses invited, 165 agreed to participate. Seven questionnaires were excluded because of incomplete responses, and the final analysis was conducted with 158 nurses. The participation rate was 63.2%.

### Data collection instruments

Three instruments were used for data collection. The first was a Personal Information Form developed by the researchers based on a review of the literature, including nurses’ sociodemographic characteristics. The second was the Yakut Moral Intelligence Scale, developed by Yakut et al. (2021). The third was the Attitude towards Medical Errors Scale, developed by Güleç and İntepeler (2013) [31, 33].

### Personal information form

This form collects information about nurses, including their age, gender, marital status, level of education, years of professional experience and length of time working in intensive care units.

### Yakut moral intelligence scale

This scale, developed by Yakut et al. (2021), consists of 20 positively worded items and has a reported reliability coefficient of 0.845. Factor analysis, one of the most commonly used methods in the social sciences, was conducted to examine the scale’s construct validity. Based on the Kaiser–Meyer–Olkin (KMO) and Bartlett’s test of sphericity results, the factor structure was found to be suitable for interpretation. Internal consistency reliability was assessed using Cronbach’s alpha coefficients. The scale uses a five-point Likert scale, with response options ranging from 1 “Strongly Disagree” to 5 “Strongly Agree”. Each item is scored from 1 to 5, respectively. The total score ranges from 20 to 100, with higher scores indicating a higher level of moral intelligence. In the present study, the Cronbach’s alpha coefficient of the Yakut Moral Intelligence Scale was found to be 0.92.

### Attitude towards medical errors scale

This scale, developed by Güleç and İntepeler (2013), comprises 16 items and three sub-dimensions. The scale’s validity and reliability have been established. It is designed to evaluate healthcare professionals’ attitudes towards medical errors. The subdimensions are perception of medical errors (two items), approach to medical errors (seven items) and causes of medical errors (seven items). The scale uses a five-point Likert scale, with response options ranging from (1) “Strongly Disagree” to (5) “Strongly Agree”. Items 10 and 13 under the ‘Approach to Medical Errors’ subdimension are reverse-coded. The total scale score is calculated by dividing the total score by the number of items. Subdimension scores are calculated by dividing the total subdimension score by the number of items in that subdimension. In the present study, the Cronbach’s alpha coefficient of the Attitudes Toward Medical Errors Scale was found to be 0.94.

### Data collection

The researchers created the scales and forms used in the study using Google Forms. They visited the intensive care units and distributed the forms to nurses, ensuring they were completed correctly. Care was taken not to disrupt nursing care during the process of collecting the data. It was estimated that completing the forms would take 15–20 min. Written and verbal informed consent was obtained from the participants prior to data collection.

### Data analysis

The data obtained from the study were transferred to a computer and analysed using IBM SPSS Statistics version 22. Descriptive statistics, including mean, standard deviation, frequency, and percentage, were used to summarise the data. The normality of continuous variables was assessed using skewness and kurtosis coefficients. Values between − 1.5 and + 1.5 were accepted as indicating an approximately normal distribution. Since the study variables demonstrated normal distribution, parametric tests were used. Independent samples t-tests were performed to compare two groups, and one-way analysis of variance (ANOVA) was used to compare more than two groups. Pearson correlation analysis was conducted to examine the relationship between moral intelligence and attitudes toward medical errors. Statistical significance was accepted as *p* < .05.

### Ethical considerations

Ethics approval for this study was obtained from the Non-Interventional Clinical Research Ethics Committee of Sivas Cumhuriyet University (decision no. 2024-03/04, date 21/03/2024). In addition, permission to conduct the study was granted by Sivas Cumhuriyet University Health Services Research and Application Hospital on 24 July 2024 (No: E-93596471-010.99-451110). The study was conducted in accordance with the principles of the Declaration of Helsinki. Participation in the study was voluntary, and informed consent was obtained from all participants prior to data collection. Data were collected via an online questionnaire, with particular care taken to ensure that participation did not interfere with nursing care services or clinical responsibilities. Participants were informed about the purpose of the study, the confidentiality of the data and their right to withdraw from the study at any time without consequence. All data were collected anonymously and used solely for scientific research purposes. Data were collected anonymously through Google Forms. Participants were not asked to provide identifying information such as names, telephone numbers, e-mail addresses, or identification numbers. IP addresses were not collected during the data collection process. Access to the dataset was restricted to the researcher, and all data were stored on a password-protected computer. The collected data were used solely for scientific purposes and handled in accordance with confidentiality and data protection principles.

## Results

The aim of this study was to investigate the relationship between intensive care nurses’ moral intelligence and their attitudes toward medical errors. The findings obtained from the analyses are presented below.

As presented in Table 1, the mean age of the participants was 29.90 ± 6.71 years. Most of the nurses were female (65.8%), while 34.2% were male. The proportions of married (49.4%) and single (50.6%) participants were similar. More than half of the participants held a bachelor’s degree (53.2%), and the largest proportion had 0–5 years of professional experience (40.5%). These findings indicate that the study sample predominantly consisted of relatively young, female nurses with undergraduate-level education and early-career professional experience (Table 1).

**Table 1:** Sociodemographic characteristics of the participants

Sociodemographic characteristics	N	%	
Age mean±sd: 29.90±6.71			
Gender	Female	104	65.8
	Male	54	34.2
Marital status	Married	78	49.4
	Single	80	50.6
Educational Background	High School	26	16.5
	Associate Degree	34	21.5
	Bachelor's Degree	84	53.2
	Master's Degree	13	8.2
	Doctorate	1	0.6
Total Professional years	0-5 years	64	40.5
	6-10 years	42	26.6
	11-15 years	30	19
	16-20 years	9	5.7
	21 years above	13	8.2

Table 2 presents the descriptive findings of the study regarding nurses’ moral intelligence levels and their attitudes towards medical errors. This table shows the mean total and sub-dimension scores for the Yakut Moral Intelligence Scale (YMIS) and the Attitude towards Medical Errors Scale (AMES) for the nurses who participated in the study.

**Table 2:** Mean scores of nurses’ Yakut moral intelligence scale and attitudes toward medical errors scale (subscales and total scores)

Scales	Mean ± SD	Min–Max	Score range
**Yakut Moral Intelligence Scale (YMIS)**	79.53 ± 11.47	35–97	20–100
Empathy	19.22 ± 3.54	5–24	5–25
Conscience	21.49 ± 4.12	5–25	5–25
Self-control	17.64 ± 3.83	5–25	5–25
Kindness	21.17 ± 2.78	14–25	5–25
**Attitudes Toward Medical Errors Scale (AMES)**	3.72 ± 0.70	1.50–4.63	1–5
Perception of Medical Errors	8.50 ± 1.57	4–10	5–10
Approach to Medical Errors	23.75 ± 3.08	13–29	7–35
Causes of Medical Errors	27.29 ± 6.89	7–35	7–35

According to the study findings, the mean total score for the YMIS was found to be 79.53 ± 11.47. Given the range of scores on the scale, it can be concluded that the nurses’ level of moral intelligence is moderate to high. Examining the subdimensions revealed the highest mean scores in the conscience (21.49 ± 4.12) and kindness (21.17 ± 2.78) dimensions. Conversely, the relatively low mean score for self-control (17.64 ± 3.83) suggests that intensive working conditions may impact an individual’s ability to manage stress and maintain self-control (Table 2).

Examination of the Attitude toward Medical Errors Scale revealed that the nurses’ mean score was 3.72 ± 0.70, indicating a moderately positive attitude toward medical errors. Evaluation of the sub-dimension scores revealed that the highest mean score was for the ‘causes of medical errors’ sub-dimension, followed by the ‘approach to medical errors’ and ‘perception of medical errors’ sub-dimensions (Table 2).

Table 3 presents a comparison of the mean scores for the Yakut Moral Intelligence Scale (YMIS) and the Attitude towards Medical Errors Scale (AMES) according to nurses’ sociodemographic characteristics. In the gender-based comparison, the mean YMIS score was 80.11 ± 12.20 for female nurses and 78.42 ± 9.90 for male nurses. For the AMES, the mean score was 3.78 ± 0.70 for female nurses and 3.60 ± 0.69 for male nurses. The independent samples t-test indicated no statistically significant difference between genders for either scale (*p* > .05).

**Table 3:** Relationship between nurses’ characteristics and their scores on the Yakut moral intelligence scale and attitudes toward medical errors scale

Characteristics	*n*	%	YMIS	AMES
			Mean ± SD	Mean ± SD
**Gender**				
Female	104	65.8	80.11 ± 12.20	3.78 ± 0.70
Male	54	34.2	78.42 ± 9.90	3.60 ± 0.69
	t/p		0.877/0.407	1.473/0.874
**Education Level**				
High School	26	16.5	81.96 ± 9.08	3,74 ± 0.68
Associate degree	34	21.5	78.32 ± 9.80	3,77 ± 0.60
Bachelor’s degree	84	53.2	78.63 ± 13.03	3,63 ± 0.76
Master’s degree	13	8.2	83.30 ± 8.35	4,07 ± 0.54
Doktorate	1	0.6	85.00 ± 00.00	3,87 ± 0.01
	F/p		0.923/0.452	1.187/0.319
**Marital Status**				
Single	78	49.4	78.62 ± 10.93	3.67 ± 0.66
Married	80	50.6	80.42 ± 11.97	3.76 ± 0.74
	t/p		0.984/0.326	0.745/0.457
**Years of Professional Experience**				
0–5 years	64	40.5	80.48 ± 11.86	3.73 ± 0.78
5–9 years	42	26.6	78.57 ± 10.95	3.67 ± 0.61
10–15 years	30	19.0	80.93 ± 9.76	3.86 ± 0.61
15–20 years	9	5.7	70.11 ± 16.23	3.30 ± 0.89
≥ 20 years	13	8.2	81.30 ± 9.12	3.78 ± 0.57
	F/p		1.937/0.107	1.184/0.320

Note. Independent samples t-test was used for gender and marital status comparisons. One-way analysis of variance (ANOVA) was used for comparisons according to educational level and years of professional experience. YMIS = Yakut Moral Intelligence Scale; AMES = Attitudes Toward Medical Errors Scale. Statistical significance was set at *p* < .05

Nurses with postgraduate education had higher mean YMIS scores than other groups, according to educational status. However, ANOVA results showed no statistically significant difference in scale scores based on educational level (*p* > .05). When analysed by marital status, married nurses had slightly higher mean YMIS and AMES scores than single nurses. Nevertheless, this difference was not statistically significant (*p* > .05). Nurses with 10–15 years of professional experience had higher moral intelligence and attitudes towards medical errors scores than other groups. However, no statistically significant difference was found between years of experience and scale scores (*p* > .05). Overall, the findings suggest that gender, educational level, marital status and years of professional experience did not have a statistically significant effect on the examined scale scores (Table 3).

The analysis results revealed a strong, positive and statistically significant relationship between the two scales (*r* = .850, *p* < .001). This indicates that as nurses’ moral intelligence increases, their attitudes towards medical errors may improve positively. In other words, nurses with higher levels of moral intelligence are more likely to recognise, prevent and demonstrate a more sensitive approach to medical errors (Table 4). Sub-dimension analyses were conducted to further explore the relationship between moral intelligence and attitudes toward medical errors. All moral intelligence sub-dimensions were positively and significantly correlated with all dimensions of attitudes toward medical errors (*p* < .001). The strongest correlations were observed between conscience and perception of medical errors (*r* = .724) and between empathy and perception of medical errors (*r* = .722). Self-control demonstrated moderate-to-strong correlations with medical error perception (*r* = .674), causes of medical errors (*r* = .637), and approach to medical errors (*r* = .633). Similarly, kindness was positively associated with all dimensions of attitudes toward medical errors, with correlation coefficients ranging from 0.595 to 0.627 (Table 4).

**Table 4:** Pearson correlations between moral intelligence subdimensions and attitudes toward medical errors subdimensions

Moral Intelligence Subdimension	Medical Error Perception	Causes of Medical Errors	Approach to Medical Errors
Empathy	0.722 ***	0.698 ***	0.695 ***
Conscience	0.724 ***	0.685 ***	0.690 ***
Self-control	0.674 ***	0.637 ***	0.633 ***
Kindness	0.595 ***	0.627 ***	0.615 ***

Note. Pearson correlation coefficients are reported. ***p < .001

To further examine the factors associated with attitudes toward medical errors, a multiple linear regression analysis was performed. Moral intelligence, age, gender, marital status, educational level, and years of professional experience were entered into the model. The overall model was statistically significant (F(6,151) = 69.544, *p* < .001) and explained 73.4% of the variance in attitudes toward medical errors (R² = 0.734).

Moral intelligence was identified as the strongest predictor of attitudes toward medical errors (β = 0.849, *p* < .001). In addition, age (β = −0.199, *p* = .047) and years of professional experience (β = 0.185, *p* = .047) were significant predictors. Gender, marital status, and educational level were not significantly associated with attitudes toward medical errors (*p* > .05) (Table 5).

**Table 5:** Multiple linear regression analysis predicting attitudes toward medical errors among intensive care nurses

Predictor	B	SE	β	t	*p*
Constant	0.006	0.314	—	0.021	0.983
Moral Intelligence	0.052	0.003	0.849	20.071	< 0.001
Age	-0.021	0.010	− 0.199	-1.999	0.047
Gender	-0.087	0.063	− 0.058	-1.370	0.173
Marital Status	-0.041	0.063	− 0.029	-0.662	0.509
Educational Level	0.054	0.041	0.068	1.336	0.183
Years of Professional Experience	0.105	0.052	0.185	2.006	0.047

Note. Dependent variable = Attitudes Toward Medical Errors Scale score. B = unstandardized regression coefficient; SE = standard error; β = standardized regression coefficient. The overall regression model was statistically significant, F(6,151) = 69.544, *p* < .001, explaining 73.4% of the variance in attitudes toward medical errors (R² = 0.734; Adjusted R² = 0.723)

## Discussion

This study aimed to determine the relationship between the moral intelligence of intensive care nurses and their attitudes towards medical errors. The findings revealed that nurses demonstrated moderate-to-high levels of moral intelligence and held generally positive attitudes towards medical errors. Furthermore, a strong positive correlation was identified between moral intelligence and attitudes towards medical errors. These findings may have important implications for ethical awareness, clinical decision-making, and patient safety in nursing practice.

The finding that nurses’ moral intelligence levels were moderate to high is consistent with the ethical values, human dignity, professional responsibility and accountability that underpin the nursing profession. Moral intelligence is defined as the ability to distinguish between right and wrong, make ethical decisions, and act accordingly [7]. In contemporary healthcare systems, moral intelligence is recognised as both a personal characteristic and a professional competency that may contribute to patient safety and quality of care [6, 12].

These findings are supported by previous studies. For example, Bezanjani et al. (2019) found that nurses with higher levels of moral intelligence were more competent in ethical decision-making and placed greater emphasis on patient safety in clinical practice [21]. Similarly, Khosravani et al. (2020) found that moral intelligence among nurses was positively associated with organisational commitment and professional responsibility. Together, these findings suggest that higher levels of moral intelligence are associated with nurses’ ability to navigate ethically complex situations, particularly in high-risk environments such as intensive care units, where rapid and accurate decision-making is essential [18].

This study found no statistically significant differences in moral intelligence or attitudes towards medical errors according to gender, education level, marital status or years of professional experience. This finding aligns with prior research suggesting that moral intelligence is more closely associated with personal value systems, ethical training, and professional experiences than with demographic characteristics [20]. This suggests that moral intelligence may be a developable competency shaped by education and clinical experience rather than inherent demographic factors.

Another important finding of this study was that nurses demonstrated moderately positive attitudes toward medical errors. Positive attitudes toward recognising, reporting, and learning from medical errors are considered essential components of patient safety culture. The World Health Organization emphasises that patient safety systems should encourage learning from errors through transparent and non-punitive reporting approaches rather than individual blame [23]. Because intensive care units involve critically ill patients, complex treatment processes, and rapid clinical decision-making, maintaining positive attitudes toward medical error reporting and prevention may contribute to safer clinical practice [23].

The literature indicates that a significant proportion of medical errors arise from human factors, communication failures, heavy workloads and systemic inefficiencies [22]. Intensive care units are characterised by complex care processes and critically ill patients, making them high-risk environments for medical errors. The high level of awareness and positive attitudes observed among intensive care nurses in this study are therefore particularly important for ensuring patient safety.

One of the most important findings of this study was the strong positive association between moral intelligence and attitudes toward medical errors. Because the Attitude toward Medical Errors Scale measures nurses’ perceptions and attitudes rather than the occurrence of actual medical errors, these findings should be interpreted as indicating that nurses with higher moral intelligence tend to demonstrate greater ethical awareness and more positive attitudes toward recognising, reporting, and managing medical errors. Although these attitudes may support patient safety practices, the present findings do not indicate that moral intelligence directly reduces the occurrence of medical errors.

Consistent with this finding, previous studies have demonstrated that ethical sensitivity and moral competence are closely linked to patient safety outcomes. For instance, Afshari et al. (2025) found that greater ethical sensitivity among healthcare professionals has a positive influence on patient safety behaviours [34]. Similarly, Safavi et al. (2022) emphasised that strong ethical decision-making skills among nurses can reduce the likelihood of clinical errors, particularly in ethically challenging situations. These findings are consistent with those of the present study and reinforce the importance of integrating ethical competencies into clinical practice [35].

The sub-dimension analyses provided additional insight into the observed relationship between moral intelligence and attitudes toward medical errors. Among the moral intelligence dimensions, conscience and empathy demonstrated the strongest associations with medical error perception. This finding suggests that nurses who possess stronger ethical awareness and greater sensitivity toward others may be more likely to recognize potential risks, acknowledge medical errors, and adopt patient-safety-oriented behaviours. Although self-control and kindness were also significantly associated with all dimensions of attitudes toward medical errors, their relationships were comparatively weaker. These findings indicate that different dimensions of moral intelligence may contribute to patient safety attitudes through distinct psychological and professional mechanisms.

In addition to the correlation analysis, multiple linear regression analysis was performed to examine whether moral intelligence predicted attitudes toward medical errors after controlling for demographic and professional characteristics. The results demonstrated that moral intelligence was the strongest predictor of attitudes toward medical errors (β = 0.849, *p* < .001). Furthermore, age and years of professional experience were identified as significant predictors, whereas gender, marital status, and educational level were not significantly associated with attitudes toward medical errors. These findings indicate that moral intelligence contributes to attitudes toward medical errors independently of most sociodemographic characteristics and may represent an important professional competency associated with patient safety and ethical clinical practice.

The magnitude of the correlation observed in this study (*r* = .850) warrants careful consideration. One possible explanation is that both constructs were measured using self-report instruments administered simultaneously, which may have increased the likelihood of common method variance. Nevertheless, the two scales assess theoretically distinct concepts. While moral intelligence reflects ethical capacities such as conscience, self-control, and kindness, attitudes toward medical errors focus on awareness, perceptions, and responses related to patient safety and error management. Therefore, the strong association observed may indicate that ethical competencies may play a substantial role in shaping nurses’ attitudes toward medical errors. However, future studies using longitudinal designs, multiple data sources, or objective patient safety indicators are needed to further validate these findings.

The findings of the present study may also be interpreted within the context of ethical decision-making and error reporting behaviour. Intensive care nurses frequently encounter complex clinical situations that require rapid decisions under conditions of uncertainty and high patient acuity. In such circumstances, moral intelligence may support ethical decision-making by enabling nurses to evaluate the consequences of their actions, recognize ethical dilemmas, and prioritize patient welfare. Furthermore, nurses with stronger ethical awareness may be more willing to acknowledge and report medical errors rather than conceal them. Since effective error reporting systems are fundamental to learning from adverse events and preventing future incidents, moral intelligence may indirectly contribute to safer clinical practice through its influence on reporting behaviours and ethical accountability.

The relationship identified in this study should also be considered within the broader framework of patient safety culture and organizational factors. Although individual characteristics such as moral intelligence appear to influence attitudes toward medical errors, patient safety culture may also be influenced by workplace conditions and institutional culture. Intensive care units are characterized by high workloads, critically ill patients, frequent interdisciplinary interactions, and time-sensitive decision-making processes. Organizational factors such as leadership support, staffing adequacy, teamwork quality, communication practices, and the presence of non-punitive reporting systems may strengthen or weaken the impact of individual moral competencies on patient safety behaviours. Therefore, improving patient safety is likely to require not only the development of nurses’ ethical capacities but also the creation of supportive organizational environments that encourage transparency, continuous learning, and open communication regarding medical errors.

The findings of this study also emphasise the importance of strengthening ethics education and patient safety training within nursing curricula. Incorporating ethical decision-making, professional responsibility and patient safety principles into nursing education programmes could boost nurses’ moral intelligence. Furthermore, establishing institutional training programmes that foster a culture of safety and ethical awareness could help reduce medical errors.

The working conditions in intensive care units also play a critical role in ensuring patient safety. A high workload, long working hours and a stressful environment may negatively influence nurses’ attention and performance. Therefore, appropriate staffing levels, improved working conditions and a supportive organisational environment are essential for minimising medical errors. Patient safety culture may be influenced not only by individual awareness but also by organisational policies and system-level interventions.

This study has several limitations. Firstly, it was conducted in a single university hospital, meaning the findings cannot be generalised to all nurses. Additionally, the data were collected using self-report measures, which may have introduced response bias. Future studies should be conducted with larger and more diverse samples across multiple institutions. Furthermore, multicentre studies examining the relationships between moral intelligence, ethical sensitivity and patient safety culture would make valuable contributions to the literature.

In conclusion, this study revealed a significant correlation between the moral intelligence of intensive care nurses and their attitudes towards medical errors. Those with higher levels of moral intelligence appear to be more sensitive to patient safety issues. Therefore, greater emphasis should be placed on ethics and patient safety in nursing education, and institutional programmes that promote ethical awareness and a culture of safe care should be implemented. In addition, attitudes toward medical errors were assessed using a self-report instrument rather than objective measures of medical error occurrence. Therefore, the findings should be interpreted as reflecting nurses’ attitudes toward medical errors rather than actual medical error events.

## Conclusion

This study revealed a strong, positive correlation between the moral intelligence of intensive care nurses and their attitudes towards medical errors. The findings suggest that higher moral intelligence scores are positively associated with greater awareness, sensitivity and proactive attitudes toward recognising, preventing and managing medical errors. Ethics education and professional development in ethical decision-making may contribute to the development of nurses’ moral intelligence; however, this relationship should be investigated through longitudinal and intervention studies. Additionally, fostering a patient safety culture that encourages open communication, error reporting and non-punitive approaches is essential for supporting patient safety and fostering positive attitudes toward recognising and reporting medical errors.

Healthcare institutions should also support nurses by providing adequate staffing levels, improving working conditions and implementing organisational policies that prioritise ethical practice and patient safety. Further research is recommended to explore this relationship in different clinical settings and with larger, multicentre samples, as well as examining the effects of interventions aimed at improving moral intelligence on patient safety outcomes. Due to the cross-sectional design of the study, causal inferences cannot be drawn from the observed relationships.

## Data Availability

No datasets were generated or analysed during the current study.
